# Nanopiezoelectric Devices for Energy Generation Based on ZnO Nanorods/Flexible-Conjugated Copolymer Hybrids Using All Wet-Coating Processes

**DOI:** 10.3390/mi11010014

**Published:** 2019-12-20

**Authors:** Yu-Ping Lee, Chieh-Chuan Lin, Chih-Chung Hsiao, Po-An Chou, Yao-Yi Cheng, Chih-Chen Hsieh, Chi-An Dai

**Affiliations:** 1 Department of Chemical Engineering, National Taiwan University, Taipei 10617, Taiwan; d98524020@ntu.edu.tw (Y.-P.L.); R97524002@ntu.edu.tw (C.-C.H.); jjoe100892@gmail.com (P.-A.C.); 2 Institute of Polymer Science and Engineering, National Taiwan University, Taipei 10617, Taiwan; R97549027@ntu.edu.tw; 3 Department of Molecular Science and Engineering, National Taipei University of Technology, Taipei 10608, Taiwan; ycheng@ntut.edu.tw

**Keywords:** ZnO nanorods, conducting polymer, nanocomposite materials, wet chemical method, piezoelectric effect

## Abstract

In this study, nanopiezoelectric devices based on ZnO nanorod array/conducting polymers are fabricated for wearable power generation application. To replace the inorganic rigid indium-tin oxide (ITO) conducting coating commonly used in the nanogenerator devices, a series of flexible polyaniline-based conducting copolymers underlying the perpendicularly-oriented ZnO nanorod arrays has been synthesized with improved electric conductivity by the copolymerization of aniline and 3,4-ethylenedioxythiophene (EDOT) monomers in order to optimize the piezoelectric current collection efficiency of the devices. It is found that significantly higher conductivity can be obtained by small addition of EDOT monomer into aniline monomer solution using an in-situ oxidative polymerization method for the synthesis of the copolymer coatings. The highest conductivity of aniline-rich copolymer is 65 S/cm, which is 2.5 times higher than that for homopolymer polyaniline coating. Subsequently, perpendicularly-oriented ZnO nanorod arrays are fabricated on the polyaniline-based copolymer substrates via a ZnO nanoparticle seeded hydrothermal fabrication process. The surface morphology, crystallinity, orientation, and crystal size of the synthesized ZnO nanorod arrays are fully examined with various synthesis parameters for copolymer coatings with different monomer compositions. It is found that piezoelectric current generated from the devices is at least five times better for the device with improved electric conductivity of the copolymer and the dense formation of ZnO nanorod arrays on the coating. Therefore, these results demonstrate the advantage of using flexible *π*-conjugated copolymer films with enhanced conductivity to further improve piezoelectric performance for future wearable energy harvesting application based on all wet chemical coating processes.

## 1. Introduction

The research of developing ZnO nanomaterials has recently attracted numerous interests, by the discovery of its particular optoelectronic [1,2,3], piezoelectric [4,5,6], and biocompatible [7,8,9] properties, which grant this nanomaterials great possibility in electronics [10,11,12,13,14,15,16,17,18], biomedical devices [19,20,21], and power generation application [22,23,24,25,26,27]. Especially, the piezoelectric property of ZnO nanorods makes this versatile material for nanotechnology applications, which include piezoelectric field-effect transistors [28,29,30] and diodes [31,32,33]. ZnO nanorods have been reported that can be used to generate rectifying piezoelectric current, which results from their combined semiconducting and piezoelectric characteristics upon the application of external forces [34,35,36,37], such as pushing, bending, vibration or rolling force, etc. The piezoelectric effect of the ZnO nanostructure is due to its wurtzite hexagonal structure (space group P6_3mc_), where the Zn^2+^ and O^2−^ ions form a tetrahedral coordination ionic crystals that lacks a center of symmetry, with lattice parameters of a = 0.325 nm and c = 0.521 nm [38,39,40,41]. In addition, ZnO is a crystal with polar surfaces, such as a positive polar plane (0001) rich in Zn^2+^ ions and a negative polar plane ($000\bar{l}$) rich in O^2−^ ions, where the interaction of the polar surfaces produces the formation of a variety of unique nanostructures, such as nanospring [42,43,44,45,46,47,48,49], nanohelices [50,51], etc. The ZnO nanomaterial application versatility is certainly relied on the diversification of ZnO crystal morphology, including rod [52,53], wire [54,55], and particle [56,57], with different size, shape, crystal density, crystallinity, and crystal orientation. Recent literature demonstrates that a model has been established to present a theory that cations and anions were displaced in different directions under the application of external electric field or mechanical forces [58,59]. The displacement of ZnO nanorods generated by external forces results in the lattice deformations for ZnO ionic crystal, which lacks a center of symmetry, inducing the piezoelectric effect. Wang et al [4] reported the study of converting the mechanical energy in nanoscale into electrical energy by applying a dragging force on the ZnO nanowire arrays, therefore establishing the method of nanogenerators (NGs) for collecting mechanical energy. Thereafter, in the field of nanopiezotronics there have been various attempts to implement and utilize the semiconducting/coupled piezoelectric properties of ZnO nanostructure for novel application [60,61,62]. It is well-known that the nanopiezotronic devices composed of highly oriented ZnO nanorod arrays exhibit superior performance, as compared to ill-aligned ZnO nanorod structure [63,64].

In this study, we present a novel ZnO nanopiezoelectric devices based on a hydrothermal method to synthesize ZnO nanorod arrays on polyaniline-based conducting polymer coatings with different electric conductivities. To replace the commonly used indium-tin oxide (ITO) conducting coating in the nanogenerator devices, a series of the conducting polymer coatings underlying the perpendicularly-oriented ZnO nanorod array has been synthesized by the copolymerization of 3,4-ethylenedioxythiophene (EDOT) and aniline at different monomer compositions in order to optimize the piezoelectric current collection efficiency of the devices for the new all wet chemical coating processes. Subsequently, perpendicularly oriented ZnO nanorod arrays are fabricated on the various conducting copolymer coatings thus made via a seeded hydrothermal fabrication process. The surface morphology, crystallinity, orientation, growth rate, rod diameter, and rod length of ZnO nanorod arrays are fully examined with various synthesis parameters for different copolymer coatings with details described below.

## 2. Experiment Section

### 2.1. Preparation of Conductive Copolymer of EDOT (3,4-Ethylenedioxythiophene) and Aniline 

The materials used in this study were purchased from commercially available sources and used as received. Polyaniline-based copolymer films were prepared via spin coating their precursor solution on glass substrates then heated at 110 °C for the in-situ chemical oxidative copolymerization. The preparation procedure of the precursor solution for copolymerization is shown as follows. Fe(OTs)_3_ and imidazole were separately dissolved in methanol in two different flasks. The two monomers, EDOT and aniline, in different composition ratios shown in Table 1, were added into the imidazole/methanol solution followed by thoroughly mixing of the mixture. The initiator solution of Fe(OTs)_3_/methanol was then added into the monomer mixture solution of EDOT/aniline/imidazole/methanol to make a 15 wt% monomer/methanol precursor solution. A copolymer precursor film can be made by spin coating the corresponding precursor solution on a pre-cleaned glass substrate with the coating condition of 1100 rpm for 10 s, followed by transferring the coated substrates in an oven heated at 110 °C for 30 min for the copolymerization. After the copolymerization, the synthesized polyaniline-based copolymer coatings were rinsed with methanol repeatedly to remove unreacted monomers. The composition of the precursor solutions for synthesizing each polyaniline-based copolymer coatings is listed in Table 1.

### 2.2. Deposition of ZnO Seed Layer on Polyaniline-Based Copolymer Coating

In order to ensure the fabrication of well-organized and perpendicularly oriented ZnO nanorod arrays on the surface of the synthesized conducting copolymer coatings, prior to the hydrothermal synthesis method for ZnO nanorod fabrication, a seed layer of crystalline ZnO nanoparticles needs to be deposited on the conducting copolymer coatings. The procedure for depositing the ZnO seed layer on polyaniline-based copolymer coating by a spin-coating method can be divided into two steps. Step one is to prepare a ZnO nanoparticle suspension solution by mixing zinc acetate dihydrate and sodium hydroxide (NaOH) in methanol. Zinc acetate dihydrate (0.11 g) and NaOH pellets (0.03 g) were dissolved in 50 g and 25 g of methanol, respectively. The NaOH methanol solution was added drop by drop into the zinc acetate/methanol solution for the formation of ZnO nanoparticle seeds. After refluxing the mixed solution at 60 °C for 2 hours, a transparent ZnO nanoparticles suspension solution could be obtained. In the second step, ZnO nanoparticles can be deposited on the copolymer coatings by spin coating the seeds solution three times. Between each spin coating, the coated substrate was heated at 140 °C in an oven for 10 min to ensure the adherence of the particles on the substrate surface. The thickness of ZnO seed layer, which was measured by using a profilometer, was about 200 nm.

### 2.3. Hydrothermal Growth of ZnO Nanorods

A hydrothermal fabrication process was used for fabricating ZnO nanorod arrays on a seed coated copolymer film substrate. The seeded copolymer coatings were dipped face down in an aqueous solution of zinc nitrate hexahydrate (0.04 M) and hexamethylenetetramine (0.04 M). The solution was sealed in a bottle and heated to 95 °C for 6 hours. After the reaction, the substrate was rinsed with DI water to remove any residual chemicals on the surface and then dried in air. 

### 2.4. Field Emission Scanning Electron Microscopy (FE-SEM)

The surface morphology of ZnO nanorod array/polyaniline-based copolymer coatings was analyzed by using field scanning electron microscopy (FESEM, FEI Nova230, FEI, Hillsboro, OR, USA). The samples were coated with a thin coating of platinum via a sputtering ion coater on a stub, where the coating time was 90 s and the applied current was 20 mA, to avoid any charging effect of samples during SEM measurements.

### 2.5. Thickness Measurement

The thickness of the ZnO seed layer on the substrate was measured using a profilometer (Microfigure Measuring Instrument-Surfcorder ET3000, Kosaka Laboratory Ltd., Tokyo, Japan).

### 2.6. X-ray Diffraction (XRD) Analysis

The crystalline structure and the overall crystallinity of the deposited ZnO seed layers and the subsequently fabricated ZnO nanorods were investigated by the X-ray diffraction (XRD, X’PERT, Malvern Panalytical Ltd., Malvern, UK) method with a monochromatic Cu K*α* radiation at 40 KV.

### 2.7. Conductive Atomic Force Microscopy (c-AFM) for Piezoelectric Current Measurement

The measurement of piezoelectric current was based on a report by Wang et al [4]. The piezoelectric current generated from the ZnO nanorod array/copolymer hybrid coatings upon the application of an external force from the AFM stylus tip on ZnO nanorods was measured by conductive AFM (AFM, NanoNavi E-sweep, Seiko, Japan) in the contact mode using a Si cantilever coated with an Au layer. The calibrated normal spring constant of the cantilever was 0.25 N/m. In the conductive AFM measurement, the normal force between the sample surface and tip was set at a constant of 5 nN. The tip’s height was automatically adjusted in keeping with the surface topography and a constant contacting force was maintained when the tip was scanned across the ZnO nanorod arrays. A silver paste was used to make an electrical connection between polyaniline-based copolymer coating and conductive AFM measurement circuit. No bias voltage was applied during the conductive AFM measurement for measuring piezoelectric current.

### 2.8. Fabrication of Large Area NG Devices for Piezoelectric Current Measurement

To demonstrate the feasibility for future application, large area (2 cm by 2 cm) piezoelectric nanogenerator devices based on the current ZnO nanorod array/copolymer coatings were fabricated. The piezoelectric nanogenerator was further assembled by covering an Au foil as one electrode on the surface of ZnO nanorod arrays and applying silver paste on the copolymer coating as the other. The contact electrodes (Au and Ag) at both ends of the semiconducting ZnO nanorod layers were Schottky contacts in accordance with the general requirements for their piezoelectric property measurements. The piezoelectric current was measured using an ammeter (CH Instruments CHI 4052A Electrochemical Analyzer, CH Instruments Inc., Austin, TX, USA) when an external force was applied by rubbing the Au foil layer on the NG device with a pressure roller. 

## 3. Results and Discussion

In this study, we fabricated a series of ZnO nanorod arrays on conducting copolymer coatings via a seeded hydrothermal method for uses in nanopiezoelectric device application. Polyaniline-based conductive copolymer coatings, on which ZnO nanorod arrays were grown, with different electrical conductivity and surface morphology were synthesized to study their effects on the piezoelectric properties of the resulting nanogenerator devices thus made. The piezoelectric devices investigated in this study has the following structure in the order of metal (from the conductive AFM Au tip in c-AFM or an Au foil covered on NG device)/piezoelectric ZnO semiconductor nanorod array/conductive copolymer coating /silver paste connected to the AFM machine.

Polyaniline-based conductive copolymer films with different conductivity and surface morphology are prepared by using in-situ chemical oxidation copolymerization of EDOT/aniline precursor solutions at different monomer molar ratios. Conductive polymers have the advantage of combining conventional polymer properties with electronically conductive materials, and they have received significant attention due to their low cost, low density, lightweight, and flexible properties for wearable electronic applications. de Leeuw [65] and Ha [66] et al. reported that the addition of imidazole as an inhibitor to impede the generation of monomeric and oligomeric radicals during the oxidative polymerization and to increase the overall molecular weight of the synthesized polymers and thus to enhance the conductivity and transparency of the polymerized PEDOT films. However, since aniline monomer also acts as an inhibitor for the copolymerization reaction, therefore, no additional weak base of imidazole is required for the synthesis of the polyaniline-based copolymer coating. The composition of precursor solutions and conductivity of the corresponding copolymer films thus synthesized are listed in Table 1. The copolymers shown in Table 1 are designed as EXAY, where E and A stand for EDOT and aniline, respectively, and X and Y represent the molar ratio for EDOT and aniline monomers added in the precursor solution, respectively. For example, for pure homopolymer aniline coating, the corresponding sample designation is PANI, while for A9E1, the copolymer coating corresponds to a precursor solution with 90% of aniline and 10% of EDOT monomer. For the three polyaniline-based copolymer films (PANI, A9E1, and A8E2) investigated, the conductivity of A9E1 was 65 S/cm, which was five times higher than that (13 S/cm) for PANI and 2.5 times better than that for A8E2 (25 S/cm). In order to understand the effect of monomer composition on the conductivity measured in the conductive copolymer coatings, more measurements need to be done.

**Figure 1:**
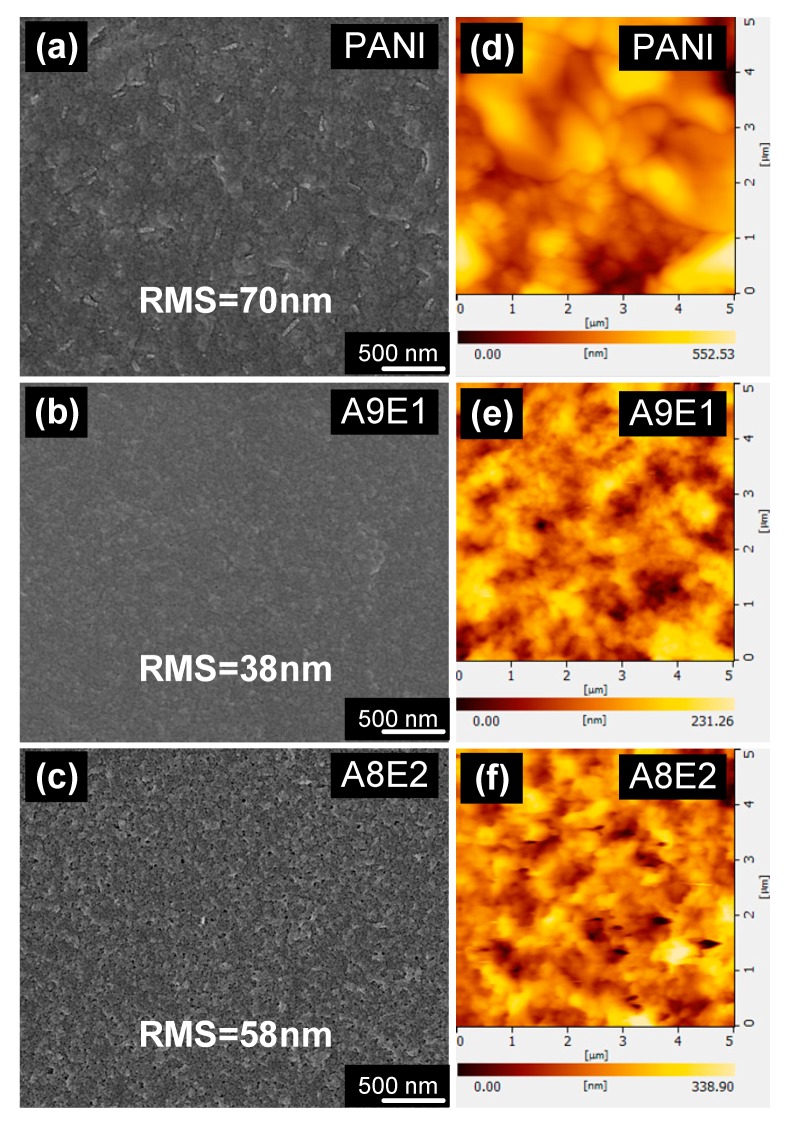
(**a**–**c**) SEM images and (**d**–**f**) atomic force microscopy (AFM) images and the measured RMS (root-mean-squared) surface roughness for the polyaniline-based conductive copolymer coatings.

The surface morphology of the synthesized polyaniline-based conductive copolymer coatings was measured by using scanning electron microscopy (SEM). Figure 1a–c show the morphology for polyaniline-based copolymer surfaces. From the SEM observation, it could be found that the homopolymer coatings show signs of uneven surface structure of white specks as well as the formation of severe cracks on the surface, which might significantly affect the roughness of the polymer films thus made, therefore, possibly the quality of the subsequently grown ZnO nanorod arrays. Notably, with a small addition of EDOT monomer in aniline-rich copolymers, the surface for the A9E1 (Figure 1b) sample appeared to show less of white spots and/or cracks compared with its homopolymer surface, indicating that the addition of EDOT might play some roles in affecting the copolymerization reaction such that its film uniformity was improved. However, with further increasing the minor monomer composition to 20% for A8E2 sample, white specks and/or cracks reappeared on their surfaces as shown in Figure 1c. Therefore, for the composition range investigated, it appeared that with the minor monomer composition at 10%, the surface uniformity of the A9E1 sample was optimized.

Based on the above SEM measurements, only qualitative information could be obtained for the surface uniformity for the copolymer coatings. In order to further investigate the monomer composition effect on the detailed surface microstructure of the coatings, atomic force microscopy (AFM) were performed. In addition, quantitative information such as surface roughness of the copolymer coatings could be attained. As shown in Figure 1d–f, the morphology and the measured surface roughness of the samples could be obtained using AFM measurements. Consistent with the SEM results, A9E1, samples with only 10% of EDOT monomer composition, show the lowest RMS (root-mean-squared) of surface roughness compared with all other samples.

**Figure 2:**
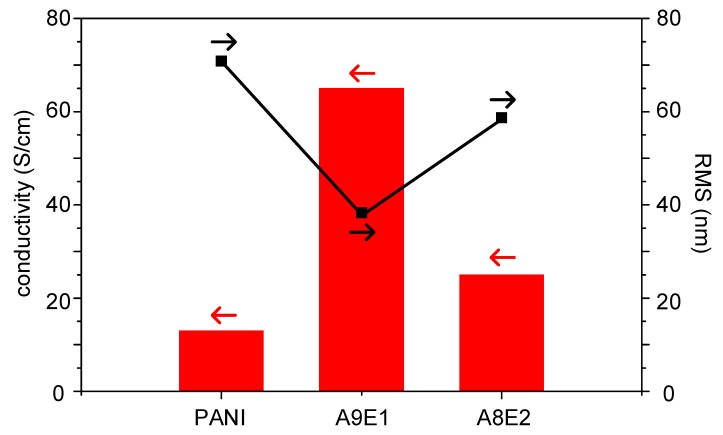
RMS surface roughness (black square) and conductivity (red bar) for polyaniline-based copolymer coatings.

Both conductivity and surface roughness results are plotted in Figure 2 for all samples. It shows that the conductivity measured in the coating increased inversely proportionally to its surface roughness value. Both properties show that with small addition of the EDOT monomer, both properties could be synergistically improved. We proposed that this enhancement effect might be due to the inhibition effect of the minor monomer addition on the copolymerization rate, which in turn increased the molecular weight of the synthesize copolymers. In addition, the reduction in the copolymerization rate ensured the formation of uniform film surface with less defects and cracks due to the increase in molecular weight of the synthesized polymers to ensure better film formation property.

**Figure 3:**
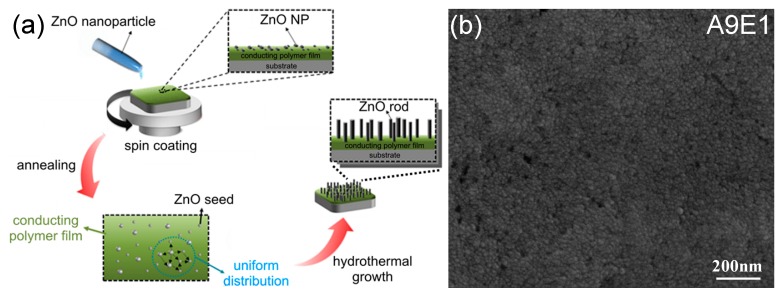
(**a**) Schematic illustration of fabrication procedure for forming ZnO seeds layer and the subsequent hydrothermal synthesis for producing ZnO nanorod arrays. (**b**) SEM images of the A9E1 copolymer coated with ZnO nanoseeds.

After polyaniline-based copolymers were coated, a seeded hydrothermal method was adopted to fabricate the ZnO nanoparticle seed coating followed by the formation of perpendicularly oriented ZnO nanorod arrays on the seed coating. Figure 3a is the schematic illustration of the seeded hydrothermal fabrication procedure for the formation of the ZnO nanorod arrays. After the deposition of ZnO nanoparticle seed layers on the conducting copolymer coatings, Figure 3b show the SEM images of A9E1 surfaces after the seed layer coating. Compared with A9E1 before the seed layer coating with images shown in Figure 1b, the sample after a seed coating exhibited the presence of ZnO nanoparticles with a uniform particle size of 20 nm coated on the film surface to ensure the subsequent growth of ZnO nanorod arrays.

**Figure 4:**
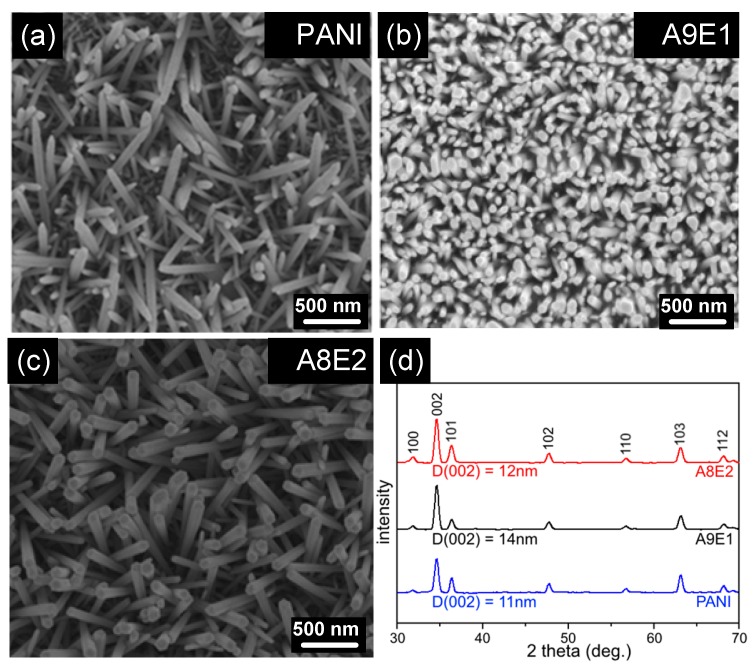
Top-view SEM images of ZnO nanorod array nanogenerator (NG) samples fabricated from (**a**) PANI, (**b**) A1E9, and (**c**) A8E2 coatings. (**d**) The XRD spectrum for the three ZnO nanorod NG coatings. The grain size D of the (002) diffraction peak for the NG coatings is calculated from measuring the full width at half maximum (FWHM) of the (002) diffraction peak using the Scherrer equation.

The hydrothermal synthesis process for the growth of ZnO nanorods has been previously developed in order to fabricate the well-organized perpendicularly-oriented ZnO nanorod arrays on glass substrate [6]. An optimized synthesis condition was adopted in this study in terms of improving growth rate, rod diameter, and rod length of ZnO nanorods that were thus synthesized. Therefore, once the ZnO seed layer was successfully coated, the seeded copolymer coatings were immersed in an equal molar aqueous solution of zinc nitrate hexahydrate (0.02 M) and hexamethylenetetramine followed by placing the solution in a heated oven at a temperature of 95 °C for 6 hours. After completing the hydrothermal synthesis process, the coating was then rinsed with DI water several times to remove any residual chemicals or ions left in the coating. Figure 4a–c shows the top view SEM images for the coatings after the hydrothermal synthesis, which all exhibit the growth of perpendicularly oriented ZnO nanorod arrays. However, the degree of uniformity for the alignment was somewhat different among samples. It can be shown that A9E1 (Figure 4b) sample, which had the lowest roughness for their copolymer coatings, a densely populated but even distribution of ZnO nanorod arrays was grown. Since the A9E1 copolymer coating had a lower roughness value, therefore, uniform and better perpendicular-aligned ZnO nanorod arrays could be subsequently synthesized. However, with higher roughness for the initial homopolymer coatings and copolymer coatings of 20% of EDOT monomer, the subsequently grown ZnO nanorod arrays were less populated, leading to a less perpendicular alignment as less populated ZnO nuclei had more freedom in lateral space to grown their nanorod sideway, leading to the formation of clumps of ZnO nanorod structure with random alignment relative to the coating surface. In addition, the width of nanorod thus synthesized on polyaniline-based copolymer coating with rough surface appeared to be thicker in width than those on smoother surface. Since copolymer coatings with smoother surface had higher density of ZnO nanoseeds, therefore, it would lead to the formation of more perpendicularly oriented nanorod arrays with narrower width of nanorods. However, for copolymer coatings with large roughness, it would lead to lower density of ZnO nanoseed formation, therefore, less density of nanorods nucleated on the surface, which in turn increases the width of each nanorod thus formed.

The X-ray diffraction technique can also be used to further measure the crystallinity and the grain size for the synthesized nanorod arrays on copolymer coatings with different surface roughness. As shown in Figure 4d, the XRD spectrum for all samples exhibited a characteristic diffraction pattern for ZnO nanorods with a very strong (002) diffraction peak and a relatively weak (103) peak, indicating the vertical growth of ZnO nanorods. In particular, ZnO NG devices with smooth surface and high conductivity of copolymer coatings of A9E1 exhibited higher and sharper (002) diffraction peak, demonstrating that a smoother copolymer provided better surface for seed layer coating, therefore, denser subsequently grown ZnO nanorod with high crystallinity and larger grain size. The average crystallite size of the ZnO nanostructures along the (002) plane can also obtained by the following Scherrer equation

D = 0.9λ/B·cos*θ*,

where D is the grain size, λ is 1.5 Å, and B is the full width at half maximum (FWHM) of the corresponding diffraction peak. The average crystallite size of the ZnO nanostructures along the (002) plane of PANI, A9E1, and A8E2 is 11 nm, 14 nm, and 12 nm, respectively, confirming that better crystalline structure of ZnO nanorods could be synthesized with a smoother underlying copolymer surface.

**Figure 5:**
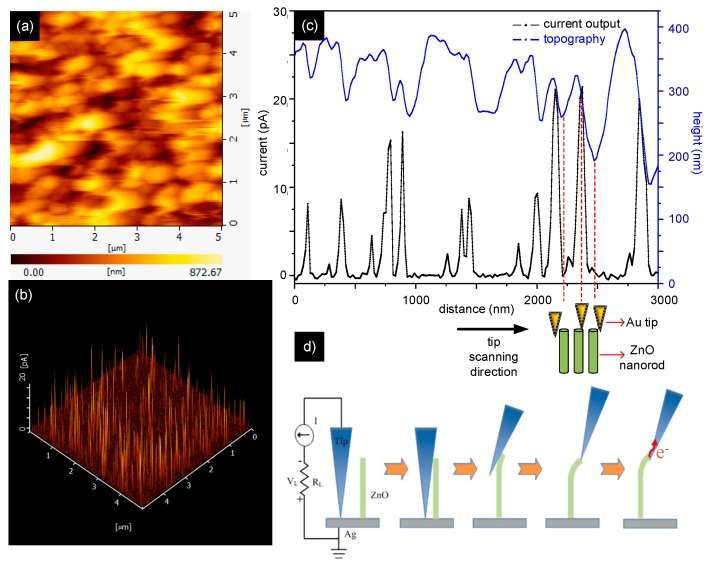
(**a**) Two-dimensional topological image and (**b**) the corresponding piezoelectric output current density image of an A9E1 NG device scanned by using an Au-coated c-AFM tip. (**c**) The topography and piezoelectric output current density are simultaneously recorded during a one-dimensional tip scanning. By comparing the two signals closely, there is a delay for the piezoelectric current, indicating that there is no electric power output when the tip first contacts ZnO nanorod. However, a sharp output current is generated when the top is about to leave the contacts with ZnO nanorod. (**d**) The schematic representation of the Au coated tip movement with ZnO nanorods is plotted.

Figure 5a,b shows simultaneous scanning topography plot and current-output profile, respectively, for a line scan of a c-AFM Au coated tip across the ZnO nanorod array of the A9E1 sample. The measurement method was originally developed by Wang and Song in 2006 [4]. For the piezoelectric current measurement c-AFM is operated in contact mode to deflect the aligned ZnO nanorods. As shown in Figure 5b, multiple sharp piezoelectric output current signals with an average peak current at about 10 pA were observed as the tip was scanned across the ZnO nanorods. Therefore, a piezoelectric current could be induced without any external application of bias voltage (self-biased) by rubbing and bending ZnO nanorod NG device. Figure 5c shows the relationship between surface height and output current and shows a high correlation between them, a simultaneous sharp signal could be observed at the position of the ZnO nanorod and induced corresponding piezoelectric current in c-AFM scanning. Specifically, as the AFM stylus began to bend the ZnO nanorod, no piezoelectric current was detected. The induced current was observed as the bending of the nanorod reached its extreme. As the ZnO nanorod was disengaged from the c-AFM stylus, the induced piezoelectric current decreased to zero, demonstrating that the induced piezoelectric current could be observed toward the end of each tip scanning over the ZnO nanorod. A schematic diagram of the piezoelectric current generation process is shown in Figure 5d.

**Figure 6:**
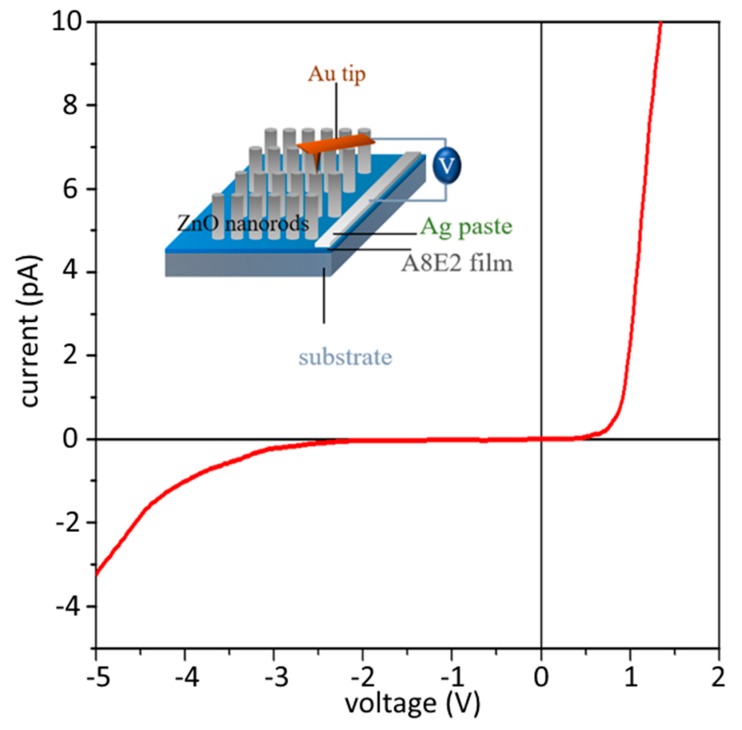
The I–V characteristics of an A8E2 sample measured by using c-AFM with an Au coated tip. The device shows the characteristic electronic property of a Schottky barrier, which exhibits a rectifying current upon the application of the forward and reverse voltage (bias).

In our current study, a p-type copolymer coating was replaced with a conventionally used n-type ITO coating as the conductive substrate in the devices. It is important to show that the overall device still exhibited a Schottky barrier, which is critical to provide the necessary piezoelectric property upon the application of external forces on the ZnO nanorod arrays. Figure 6 shows the I–V characteristics of an A8E2 sample measured by using c-AFM with an Au coating tip. The device exhibited a characteristic rectifying junction in which the current rose exponentially under a forward bias and decreased to nearly zero under a reverse bias. Therefore, the device with a p-type copolymer coating exhibited the necessary piezoelectric property as a Schottky junction. Therefore, a piezoelectric current could be induced without any external application of bias voltage (self-biased) by rubbing and bending the ZnO nanorod NG device.

**Figure 7:**
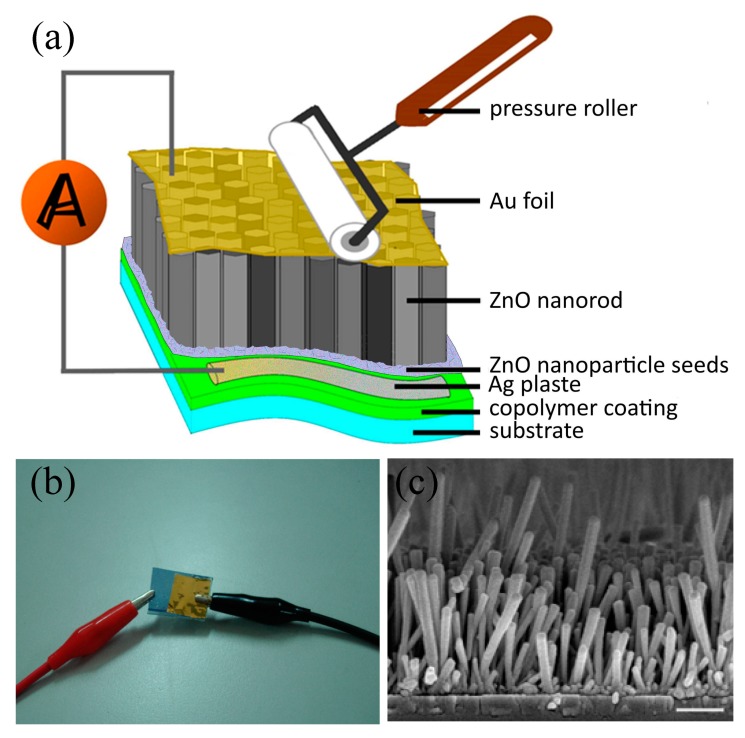
(**a**) The schematic illustration of a ZnO NG device. (**b**) An actual photo picture of a NG device, and (**c**) the cross-sectional SEM image of a piezoelectric device.

To further demonstrate the feasibility of applying our current design into practice for future application, a large area piezoelectric nanogenerator (2 cm by 2 cm) sheet was fabricated with the device structure shown in Figure 7a. As one electrode, an Au-coated polymer film was covered on the piezoelectric devices, while an Ag layer was applied on the copolymer coating as the other electrode (Figure 7b). This large area nanogenerator device was rubbed against with each by the application of external force exerted from the rolling motion of a pressure roller. An ammeter, which was connected with the electrodes, could be used to measure periodic current signal thus produced from the rolling motion. The corresponding cross-sectional SEM image of piezoelectric nanogenerator (NG) device is shown in Figure 7c.

**Figure 8:**
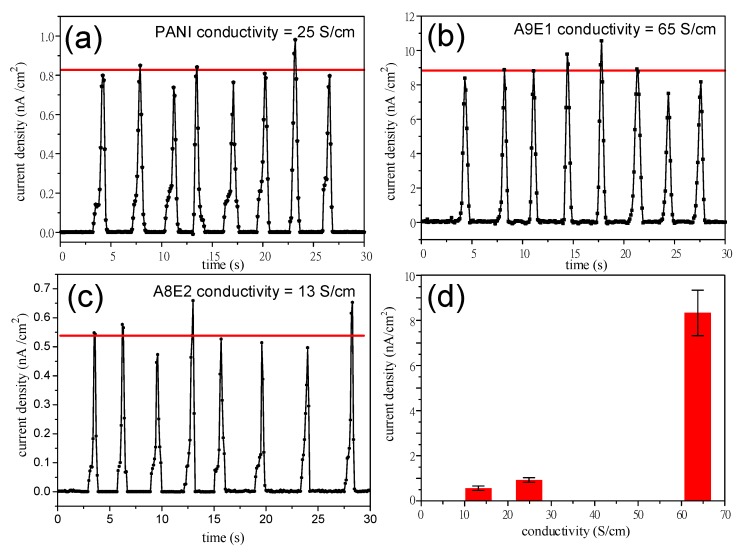
The piezoelectric current induced by pressure roller rubbing is plotted as a function of duration of rubbing time for the large area polyaniline-based NG devices. (**a**) PANI, (**b**) A1E9, and (**c**) A8E2. (**d**) The piezoelectric current density (nA/cm^2^) of the device is plotted as a function of the electrical conductivity (S/cm) of copolymer coatings. A non-linear relationship can be found between the copolymer coating conductivity and the corresponding induced piezoelectric current density.

As shown in Figure 8, the piezoelectric current generated by the rolling motion of the Au film coating was plotted as a function of time during which large area polyaniline-based piezoelectric nanogenerator devices were rubbed against a pressure roller. Note that the peak piezoelectric current generated from these large area devices was in the range of nA, which was at least 100 folds larger than those measured in the previous conducting AFM measurement in which the peak piezoelectric current thus produced was in the range of pA due to only single ZnO nanorod bending with a force exerted from the AFM Au scanning tip. The average peak piezoelectric current generated from PANI and A8E2 devices with lower conductivity and less uniform ZnO nanorod array was around 0.5 nA/cm^2^ (Figure 8a) and 0.8 nA/cm^2^ (Figure 8c), while that for A9E1 device was markedly larger at a value of 8.0 nA/cm^2^ (Figure 8). As shown in Figure 8d, the peak piezoelectric current was directly proportional to the increase in the conductivity of the underlying charge collection conductive copolymer coatings. Therefore, the combined results shown above demonstrated that an improved conductivity and uniform coating surface produced from the controlled copolymerization reaction for the subsequent formation of dense ZnO nanorod arrays with improved crystallinity were critically important in enhancing the overall piezoelectric performance of the nanogenerator devices thus produce [67,68].

## 4. Conclusions

In this study, we successfully fabricated well-organized perpendicularly aligned ZnO nanorod arrays on various polyaniline-based conducting copolymer coatings and utilized the coupled piezoelectric-semiconducting properties of ZnO nanorods and improved charge-collection performance of the conducting copolymer coatings to fabricate a series of piezoelectric current-generating devices. The active piezoelectric hybrid coatings could be produced by sequential solution-based manufacturing processes of coating conducting polymers, ZnO nanoparticle seeds, and hydrothermal synthesis of ZnO nanorod arrays, facilitating future wearable application based on all wet-chemical methods. The enhancement in the measured conductivity for the copolymer coatings with a small addition of EDOT monomer into aniline might be due to the minor monomer inhibition effect on the copolymerization rate, which enhances the molecular weight of synthesized conductive copolymer coatings. In addition, the reduction in the copolymerization rate ensured the formation of uniform film surface with less defects, leading to an enhancement in the subsequent growth of dense and well-organized ZnO nanoarrays with better crystallinity for piezoelectric performance improvement. In addition, a larger area piezoelectric device based on the current method was also fabricated to demonstrate its feasibility for future application. In conclusion, a facile all wet-chemical fabrication method was developed in order to produce nanogenerators with improved piezoelectric performance for future wearable energy harvesting applications.

## Figures and Tables

**Table 1:** The composition of precursor solutions and the conductivity of the corresponding copolymer films thus synthesized and their sample designation.

Sample	Fe(OTs)_3_/Monomers	Aniline: EDOT	Roughness (RMS/nm)	Conductivity (S/cm)
A10E0 (PANI)	1.75	10:0	70.86	13
A9E1	1.75	9:1	38.30	65
A8E2	1.75	8:2	58.69	25
